# Oesophageal safety of high and very high power short duration pulmonary vein isolation: a randomized comparison of the 50 W and 90 W power settings—the HPSD oesophagus study

**DOI:** 10.1093/ehjopen/oeag041

**Published:** 2026-02-25

**Authors:** Ferenc Komlósi, Klaudia Vivien Nagy, Péter Perge, Zoltán Salló, István Osztheimer, Edit Tanai, Patrik Tóth, Gábor Orbán, Arnold-Béla Ferencz, Márton Boga, István Hizoh, Veronika Papp, István Hritz, Attila Szijártó, Melinda Boussoussou, Béla Merkely, László Gellér, Nándor Szegedi

**Affiliations:** Heart and Vascular Center, Semmelweis University, Varosmajor u. 68., 1122 Budapest, Hungary; Heart and Vascular Center, Semmelweis University, Varosmajor u. 68., 1122 Budapest, Hungary; Heart and Vascular Center, Semmelweis University, Varosmajor u. 68., 1122 Budapest, Hungary; Heart and Vascular Center, Semmelweis University, Varosmajor u. 68., 1122 Budapest, Hungary; Heart and Vascular Center, Semmelweis University, Varosmajor u. 68., 1122 Budapest, Hungary; Heart and Vascular Center, Semmelweis University, Varosmajor u. 68., 1122 Budapest, Hungary; Heart and Vascular Center, Semmelweis University, Varosmajor u. 68., 1122 Budapest, Hungary; Heart and Vascular Center, Semmelweis University, Varosmajor u. 68., 1122 Budapest, Hungary; Heart Institute, University of Pécs, Ifjúság útja 13., 7624 Pécs, Hungary; Heart and Vascular Center, Semmelweis University, Varosmajor u. 68., 1122 Budapest, Hungary; Heart and Vascular Center, Semmelweis University, Varosmajor u. 68., 1122 Budapest, Hungary; Department of Transplantation and Gastroenterology, Semmelweis University, Üllői út 78., 1082 Budapest, Hungary; Department of Transplantation and Gastroenterology, Semmelweis University, Üllői út 78., 1082 Budapest, Hungary; Department of Transplantation and Gastroenterology, Semmelweis University, Üllői út 78., 1082 Budapest, Hungary; Heart and Vascular Center, Semmelweis University, Varosmajor u. 68., 1122 Budapest, Hungary; Heart and Vascular Center, Semmelweis University, Varosmajor u. 68., 1122 Budapest, Hungary; Heart and Vascular Center, Semmelweis University, Varosmajor u. 68., 1122 Budapest, Hungary; Heart and Vascular Center, Semmelweis University, Varosmajor u. 68., 1122 Budapest, Hungary

**Keywords:** Atrial fibrillation, Catheter ablation, Thermal safety, High power short duration

## Abstract

**Aims:**

Point-by-point radiofrequency catheter ablation is commonly used for pulmonary vein isolation (PVI) in atrial fibrillation (AF). Very high power short duration (vHPSD) technology offers similar efficacy to high power short duration (HPSD) with reduced procedure times. A major complication of thermal ablation is atrio-oesophageal fistula (AEF), with oesophageal lesions and gastroparesis indicating increased risk. A direct comparison of HPSD and vHPSD regarding thermal safety is lacking. This randomized, single-centre study aimed to compare the thermal safety of vHPSD to HPSD and identify anatomical and biophysical predictors of thermal injury (ClinicalTrials.gov ID: NCT06617442).

**Methods and results:**

Patients undergoing first-time PVI for AF were randomized to either HPSD (50 W) or vHPSD (90 W). The study followed a non-inferiority design, with the primary endpoint being a composite of oesophageal mucosal lesion and gastroparesis, assessed via endoscopy within 15 days post-procedure. Pre-procedural computed tomography scans were analysed for anatomical risk factors. Among 100 patients (50 per group), the primary endpoint occurred in 7 (14%) of the vHPSD group and 6 (12%) of the HPSD group (estimated mean difference −2%, lower bound of one-sided 95% CI −0.13), confirming non-inferiority. A smaller inter-lesion distance on the posterior wall was associated with increased risk (3.76 mm vs. 4.16 mm, *P* = 0.042).

**Conclusion:**

Pulmonary vein isolation using 90 W (vHPSD) applications was non-inferior to the 50 W (HPSD) power setting in terms of oesophageal safety. A smaller inter-lesion distance on the posterior wall was predictive of oesophageal injury.

## Graphical Abstract

**Graphical Abstract:**
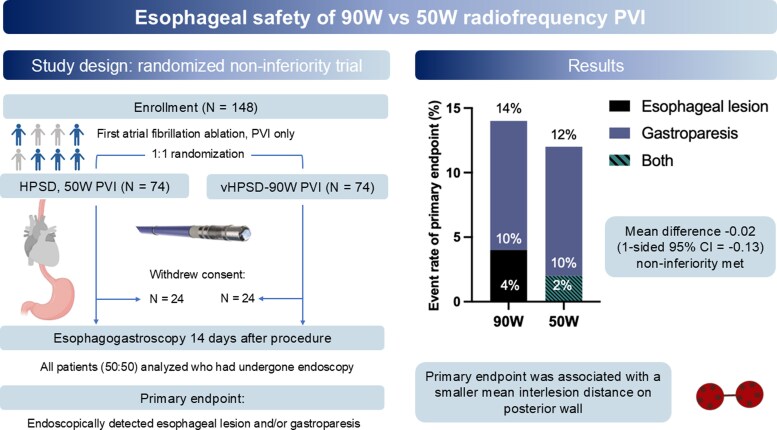

## Introduction

Pulmonary vein isolation (PVI) is the most effective method for maintaining sinus rhythm in atrial fibrillation (AF) patients^1,2^; a widely used approach is the point-by-point radiofrequency (RF) catheter ablation.^3^ Although RF ablation is effective, it is not without risk of complications. When using thermal energy sources, the close proximity of the oesophagus to the left atrial (LA) posterior wall (PW) carries the risk of oesophageal thermal injury (ETI). These lesions may evolve into atrio-oesophageal fistula (AEF), a rare but life-threatening complication with a mortality rate exceeding 60%.^4–6^ Asymptomatic thermal damage to the structures near the PW may manifest as endoscopically detected oesophageal lesions (EDELs) or signs of gastrointestinal hypomotility attributed to the thermal effect on the vagal nerve.^7^

A recent innovation in RF ablation, known as the high power short duration technique (HPSD), involves higher energy settings (40–50 W) with shorter application times. Studies examining HPSD ablation consistently report shorter procedural times with similar efficacy and safety as for the low-power long duration ablation.^8,9^ Thermal biophysics suggest that shorter application duration results in less tissue penetration and shallower lesion formation.^10^ This may lead to reduced damage to extracardiac tissues in the case of HPSD applications. The advantages of HPSD ablation have recently encouraged further (>50 W) increase of energy and reduction of application time, often termed very high power short duration ablation (vHPSD). In particular, PVI with 90 W is gaining popularity, further reducing procedural time while maintaining efficacy.^11–13^

Several studies assess the incidence of ETI following HPSD ablation with 45–50 W energy setting, using mandated post-procedural endoscopy. The reported rate of EDEL ranges from 2.5 to 16%, while gastric hypomotility was found in about 15% of the cases.^8,14–16^ However, data on the novel vHPSD energy setting remains limited. Small observational studies showed an EDEL rate of 1–2%.^11,17,18^ Importantly, none of the vHPSD studies report the incidence of gastric hypomotility. The available data suggest that vHPSD ablation may have a similar or potentially superior oesophageal safety profile than HPSD; however, a focused randomized comparison is still lacking.

The aim of this single-centre, randomized study was to compare the incidence of oesophageal lesions and gastric hypomotility in patients undergoing PVI, using 50 W or 90 W power setting. Further analysis was performed to assess the risk factors of oesophageal lesions, focusing on lesion metrics and the anatomical characteristics assessed through pre-procedural imaging.

## Methods

### Study design

**Figure 1:**
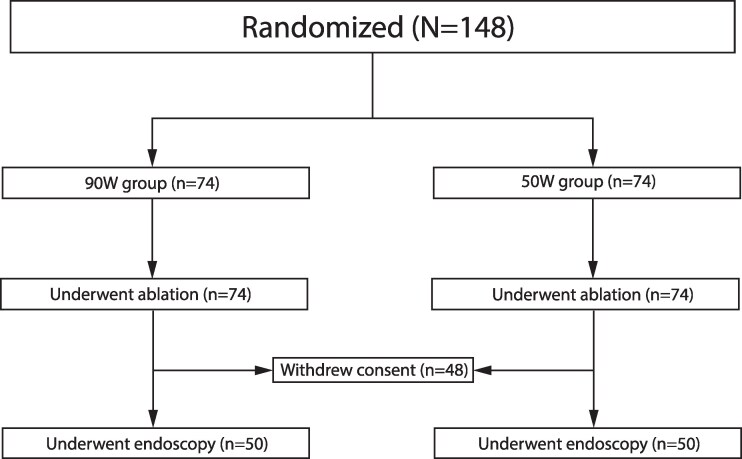
Study flowchart.

Patients with paroxysmal or persistent AF undergoing their first RF catheter ablation procedure between February 2022 and June 2024 were randomized to PVI with either HPSD (50 W) or vHPSD (90 W) power setting (*Figure 1*).

Eligible patients had symptomatic paroxysmal or persistent AF (<12 months). Patients were excluded if additional ablation beyond PVI was planned. Full details of inclusion and exclusion criteria are listed in the study protocol (see Supplementary material online, *Methods*). All participants gave written informed consent.

Patients were randomized in a 1:1 ratio to either undergo PVI with vHPSD ablation (90 W, 4 s) or ablation index (AI)–guided HPSD PVI with a 50 W power setting. Simple randomization was performed using the www.studyrandomizer.com randomization tool. Within 15 days after the index procedure, all patients underwent oesophagogastroscopy. This study was conducted in accordance with Good Clinical Practice guidelines and the Declaration of Helsinki. Ethics approval was obtained from the Hungarian National Center for Public Health and Pharmacy (Identifier: 8088-2/2022/EÜIG) (ClinicalTrials.gov ID: NCT06617442).

### Pre-procedural imaging

Prior to the ablation procedure, patients underwent contrast-enhanced computed tomography (CT) of the LA to rule out thrombus formation. A wide-detector cardiac CT scanner (GE CardioGraphe, GE Healthcare, Chicago, IL, USA) was used; patients without contraindications were given 0.8 mg of sublingual nitroglycerine. Scan parameters for coronary CT angiography were customized based on patient anthropometrics, with image acquisition conducted at a 240 ms rotation time. Systolic triggering was employed for patients with heart rates above 75 b.p.m. Iomeprol contrast agent (Iomeron 400, Bracco Ltd., Milan, Italy) was injected via antecubital venous access, using 85–95 mL of contrast agent at a flow rate of 4.5–5.5 mL/s through a four-phase protocol. Proper scan timing was ensured through bolus tracking in the LA,^19^ and images were reconstructed with statistical iterative reconstruction at level 70.^20^

To describe the anatomical relation between the LA PW and the oesophagus, several measurements were performed. In the sagittal plane of the cardiac CT scan, the length of the oesophagus adjacent to the LA wall was measured (‘oesophagus-LA contact length’). Along this segment at a superior, medium, and inferior level, the following measurements were taken in the axial plane: (i) oesophageal wall thickness (in cases where the lumen of the oesophagus was not visible, the wall thickness of the oesophagus was registered as half the total diameter); (ii) thickness of the fat pad between the oesophagus and posterior LA wall; and (iii) thickness of the posterior LA wall.^21^ Furthermore, we assessed the total distance from the endocardium to the oesophageal mucosa (sum of the oesophageal wall, fat pad, and the LA posterior wall thickness) and the total distance from the endocardium to the oesophageal adventitia (sum of the LA posterior wall and the fat pad thickness) The measurements were performed using the Philips IntelliSpace software (Portal v.6.2, Philips Healthcare).

### Catheter ablation procedure

The procedures were performed by physicians with an experience of at least 5 years and 300 cases performed. Catheter ablation was performed under conscious sedation without oesophageal temperature monitoring. After femoral venous access, double transseptal puncture was performed using fluoroscopy and pressure guidance. Heparin boluses were given to maintain an activated clotting time (ACT) above 300 s. A fast anatomical map was created using the CARTO system (Biosense Webster Inc., Diamond Bar, CA, USA) and a multipolar mapping catheter (either Lasso or Pentaray, Biosense Webster Inc., Diamond Bar, CA, USA). An irrigated, contact force (CF) sensing catheter (QDot Micro) and a steerable sheath (Agilis, Abbott Laboratories, Green Oaks, Illinois, USA) were utilized in all cases to create circumferential antral lesion set around the ipsilateral PV’s orifice. If the patient reported pain at any point, a brief pause was taken between applications, and analgesia was titrated accordingly. An inter-lesion distance (ILD) of less than 5 mm was targeted on the posterior wall in both study groups. In the HPSD group, an AI ≥ 400 on the posterior wall and AI ≥ 500 on the anterior wall were targeted. In the vHPSD group, we delivered standard 4 s applications using the 90 W energy setting (QMODE + algorithm).^12^ For each RF application on the PW, we recorded the following parameters: AI (only for HPSD), ILD, application time, average power, mean and maximal catheter tip temperature, and mean and maximal CF during energy delivery. Furthermore, the total energy delivered to the posterior wall was calculated by multiplying the mean power by the application time, totalling all applications made on the posterior wall. No additional ablation beyond PVI was performed. Patients were prescribed proton pump inhibitors (pantoprazole 40 mg daily) for 4 weeks following the procedure.

Major complications were defined as any treatment-related adverse event requiring interventional or surgical therapy or leading to a prolonged hospitalization.

### Post-ablation endoscopy

Oesophagogastroscopy was performed within 15 days of the ablation by two expert endoscopists who regularly perform diagnostic as well as interventional endoscopies, using an Olympus EVIS EXERA III, GIF-H185 device. The endoscopic endpoints were defined in consultation with two gastroenterologists in the study steering committee. Endoscopically detected oesophageal lesions were classified by severity into oesophageal erosion and oesophageal ulcer. Gastroparesis was defined as insufficient gastric emptying following overnight fasting, unexplained by previous pathologies or concomitant medication side effects. Patients with erosions were prescribed high dose proton pump inhibitors (pantoprazole 40 mg BID) for 3 months. Patients with gastric hypomotility were given standard doses of itopride for 30 days.

### Study endpoints

The primary endpoint of this study was a composite of ablation-induced EDEL and/or gastroparesis as identified at post-ablation endoscopy.

### Statistical analysis

The study was designed to demonstrate the non-inferiority of vHPSD to HPSD in terms of the incidence of the primary endpoint. Previous studies of HPSD ablation at 50 W reported EDEL rates ranging from 4.2^22^ to 10.3%.^23^ Gastric hypomotility was described in fewer studies, with an incidence ranging from 3.8^22^ to 30%.^14^ Studies using 90 W energy are limited by relatively small sample sizes but consistently show an EDEL rate of 1–2%, while the rate of gastric hypomotility has not been reported.^11,17,18^

We expected a 13% event rate in the HPSD arm and a 9% event rate in the vHPSD arm. To detect non-inferiority in absolute risk with a margin of 15%, using a one-sided significance level of 5% and a power of 90%, using 1:1 randomization, the minimal required sample size of 94 was determined. To account for the limited published data on thermal complication rates with the vHPSD energy setting and the anticipated high dropout rate for post-procedure endoscopy, we increased the planned enrolment to 150 patients, with the option of stopping early after an interim analysis of the first 100 patients who underwent endoscopy. Ultimately, the treatment arms were compared using the one-sided 95% confidence interval (CI) approach, with a non-inferiority framework. Scalar variables were expressed as mean ± standard deviation when normally distributed, while median and interquartile ranges were provided for skewed data.

Categorical data were presented as frequencies and percentages. For categorical variables, groups were compared using the χ^2^ test, while scalar data was analysed using Student’s *t*-test or Mann–Whitney *U* test, depending on whether the data was normally distributed. Statistical analysis was performed using the SciPy package^24^ in a Python programming environment.^25^

## Results

### Patient characteristics

We recruited and randomized a total of 148 patients. Forty-eight patients withdrew their consent before the endoscopy. This report presents the predefined interim analysis after 100 patients underwent post-procedure endoscopy. As the non-inferiority criterion was met, enrolment was stopped, and this analysis represents the final trial results. Baseline parameters of the study population are shown in *Table 1*. The mean patient age was 66 (55–70), with 61% paroxysmal AF; 61% of the patients were male. The most frequent comorbidities were hypertension (70%) and diabetes (20%). The mean ejection fraction was 54% (54–61%). We performed the planned interim analysis with a total of 100 patients having undergone upper gastrointestinal diagnostic endoscopy (study flowchart shown in *Figure 1*).

**Table 1:** Baseline characteristics of the two study groups

		Study group (*n* = 100)	
	All patients	90 W (*n* = 50)	50 W (*n* = 50)
Age, years	66 [55–70]	64 [55–68]	67 [55–71]
Male sex, *n* (%)	61 (61)	32 (64)	29 (58)
BMI, kg/m^2^	29 [24–33]	28 [23–33]	29 [25–33]
Paroxysmal, *n* (%)	61 (61)	26 (52)	35 (70)
Hypertension, *n* (%)	70 (70)	33 (66)	37 (74)
Diabetes, *n* (%)	20 (20)	12 (24)	8 (16)
CAD, *n* (%)	17 (17)	8 (16)	9 (18)
Stroke/TIA, *n* (%)	8 (8)	6 (12)	2 (4)
PAD, *n* (%)	3 (3)	0 (0)	3 (6)
LVEF, %	56 [54–61]	57 [55–64.8]	55.9 [46.8–65.1]

Only patients who underwent endoscopy were analysed. Categorical variables are presented as total counts and percentages, while scalars are shown as median and interquartile range (skewed data) or mean ± standard deviation (normally distributed data). *P* < 0.05 was considered statistically significant.

BMI, body mass index; CAD, coronary artery disease; TIA, transient ischaemic attack; PAD, peripheral artery disease; LVEF, left ventricular ejection fraction.

### Endoscopy findings

**Figure 2:**
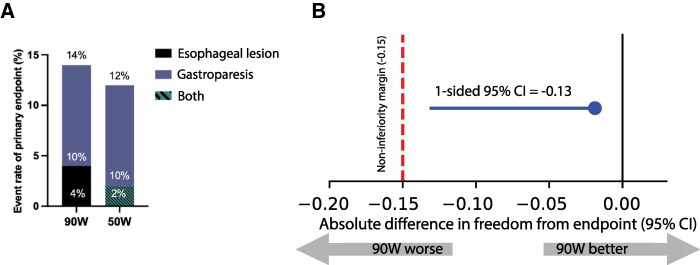
(*A*) Incidence of oesophageal lesion and gastric hypomotility in the two treatment groups. (*B*) Forest plot showing the absolute difference in success. The blue circle represents the point estimate of difference in absolute risk, while the blue bar indicates the one-sided 95% confidence interval. The predefined non-inferiority margin is shown by the red dashed vertical line. As the confidence interval does not include the non-inferiority margin, the criteria of non-inferiority are met.

**Figure 3:**
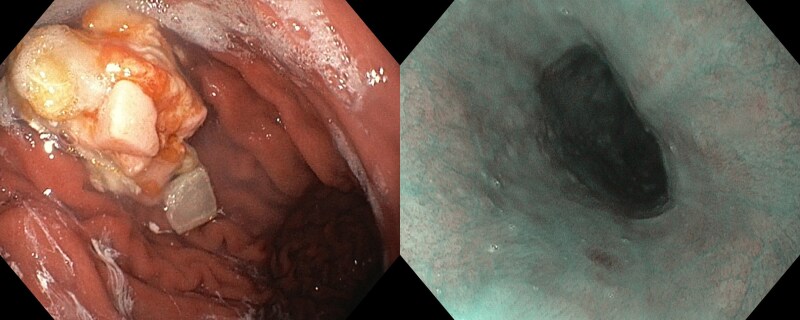
Representative images of positive endoscopic findings (endoscopically detected oesophageal lesion). Left: gastroparesis; right: erosion (shown by white arrow).

Patients underwent endoscopy at a median of 8 days after the index procedure. In the vHPSD group, we detected two erosions and five cases of gastric hypomotility. In the HPSD arm, we observed one erosion with gastric hypomotility, while five cases of isolated gastric hypomotility were registered. Overall, the incidence of the composite endpoint was 7 (14%) in the vHPSD group and 6 (12%) in the HPSD group (estimated mean difference −2% points, the lower bound of the 95% CI −0.13) (*Figure 2*). Importantly, all the detected erosions were asymptomatic and resolved without sequelae. Cases of gastroparesis were also asymptomatic and did not require fasting. Representative endoscopic images of the lesions are shown in *Figure 3*.

### Procedural characteristics

**Figure 4:**
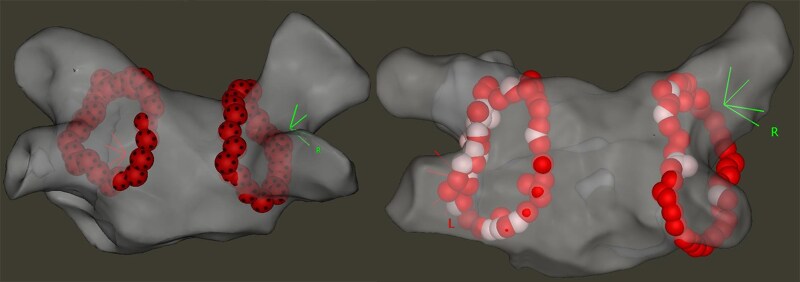
Representative images of the electroanatomical map with pulmonary vein isolation applications. Left: very high power short duration; right: high power short duration.

All pulmonary veins were successfully isolated at the end of all procedures (*Figure 4*). We found that the procedure time, RF time, and application times were shorter in the vHPSD group compared to the HPSD group (57.5 min vs. 66.3 min, *P* = 0.04, 345 s vs. 945 s, *P* < 0.001, 3.97 s vs. 10.5 s, *P* < 0.001, respectively). Detailed procedural parameters are presented in *Table 2*. The minimum ILD on the posterior wall was larger, while catheter tip temperatures and mean power were higher in the vHPSD group. No major complications were observed. Comparing procedural characteristics with regard to the endpoint, we found that the mean ILD on the posterior wall was significantly smaller in patients with post-operative oesophageal erosion or gastric hypomotility (3.76 mm vs. 4.16 mm, *P* = 0.04). Other lesion metrics did not differ across the two groups.

**Table 2:** Procedural and anatomical parameters of the two study groups

Parameter	All patients	90 W (*n* = 50)	50 W (*n* = 50)	*P*
Procedure time	60.5 [50–74]	57.5 [45–68]	66.3 [48.6–83.9]	**0**.**04**
Left atrial dwell time, min	46 [36–56]	43 [33–56.8]	48.6 [34.4–62.7]	0.24
Ablation time, min	30 [22.5–40]	28 [21–37]	34 [24.2–40]	0.17
Radiofrequency time, s	550 [336–935]	348 [257–422]	945 [627–1264]	**<0**.**001**
Applications on PW	30 [24–36]	31 [25–37]	29 [24–34]	0.21
Bilateral first-pass isolation, *n* (%)	78 (78)	36 (72)	42 (84)	0.15
Fluoroscopy time, min	7.25 [3.92–12.5]	7.25 [3.4–11.5]	7.25 [4.03–14.3]	0.42
DAP, µGym^2^	134 [76.3–304]	127 [57.0–301]	139 [84.3–303]	0.35
ED, mGy	11 [6–26.2]	10.5 [5–26]	12 [7–26.8]	0.47
Mean ILD on PW, mm	4.09 [3.71–5.35]	4.17 [3.81–5.33]	3.97 [3.69–5.38]	0.42
Minimum ILD on PW, mm	1.4 [0.9–2]	1.6 [1.2–2.15]	1.4 [0.8–1.82]	**0**.**03**
Longest application on PW, s	11.2 [4.05–18.5]	4.05 [4.03–4.05]	18.5 [15.2–21.9]	**<0**.**001**
Mean application time on PW, s	4.18 [3.97–10.7]	3.97 [3.91–3.99]	10.5 [8.33–12.7]	**<0**.**001**
Maximal catheter tip temperature on PW, °C	52 [49–57]	57 [54.5–58]	49 [48–51]	**<0**.**001**
Mean catheter tip temperature on PW, °C	42.3 [40.2–45.5]	45.8 [43.5–48.0]	40.0 [38.7–41.4]	**<0**.**001**
Mean CF during energy delivery on PW, g	15.6 [12.2–20.2]	17.7 [11.3–24.1]	14.2 [11.7–19.2]	0.15
Maximal CF during energy delivery on PW, g	83.6 [57.0–110]	79.0 [51.2–107]	88.2 [63.4–113]	0.12
Mean power, W	59.4 [46.2–82.1]	82.2 [80.2–83.0]	46.4 [45.4–47.5]	**<0**.**001**
Oesophagus-LA contact length, mm	40.0 [31.0–48.9]	40.8 [32.2–49.4]	39.1 [29.7–48.4]	0.22
PW min. thickness, mm	0.6 [0.5–0.6]	0.55 [0.45–0.65]	0.6 [0.5–0.6]	0.64
Oesophagus wall min. thickness, mm	3.81 [3.01–4.61]	3.85 [3.1–4.6]	3.77 [2.91–4.63]	0.94
Fat pad thickness, mm	0.6 [0–0.9]	0.5 [0–0.86]	0.64 [0–1.01]	0.31
Endocardium-oesophageal min. distance, mm	0.9 [0.6–1.2]	0.8 [0.6–1.2]	1 [0.6–1.2]	0.52
Endocardium-oesophageal mucosa min. distance, mm	4.41 [3.36–5.46]	4.39 [3.51–5.27]	4.43 [3.21–5.65]	0.85

Bold *P* values indicate statistically significant difference.PW, posterior wall; DAP, dose area product; ED, effective dose; ILD, inter-lesion distance; CF, contact force; LA, left atrium.

### Non-thermal complications

Two cases of pericardial tamponade occurred, one in the HPSD group and one in the vHPSD group; both were successfully managed by pericardiocentesis. Neither of the two patients underwent endoscopy. No embolic or major vascular complications occurred.

### Pre-operative computed tomography assessment

**Figure 5:**
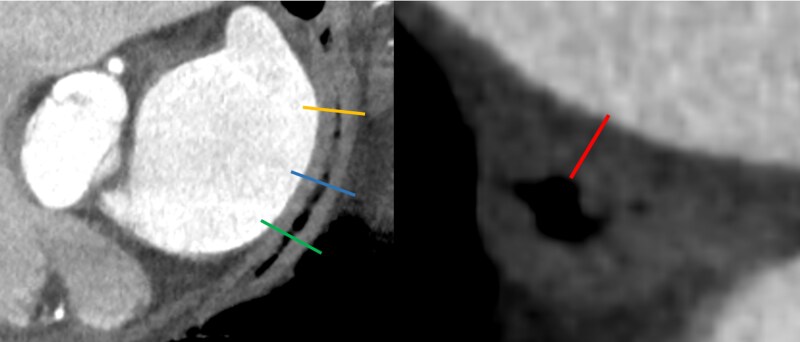
Representative computed tomography image showing the assessment of the anatomical parameters of the left atrial posterior wall and the oesophagus. Left: venous phase angiography shown in the sagittal plane through the oesophagus. Yellow, blue, and green lines show the levels of superior, mid-, and inferior measurement levels. Right: axial plane image showing the distance between the left atrial posterior wall endocardium and the oesophageal mucosa at the point of measurement (corresponding to the level of the blue line on the left figure).

Furthermore, we compared ablation and CT imaging-derived parameters between patients who did and who did not reach the primary composite endpoint (*Figure 5*). We found no difference in the oesophagus-LA contact length (40 mm vs. 36.6 mm, *P* = 0.24), LA posterior wall thickness (0.55 mm vs. 0.6 mm, *P* = 0.64), the thickness of the oesophageal wall (3.06 mm vs. 3.32 mm, *P* = 0.364), or the thickness of the fat pad between the oesophagus and the posterior wall (0.47 mm vs. 0.63 mm, *P* = 0.82). Additionally, we analysed the total distance from the endocardium to the oesophagus and from the endocardium to the oesophageal mucosa and found no significant differences (*Table 3*).

**Table 3:** Procedural and anatomical parameters compared between patients with and without the occurrence of the composite endpoint

Parameter	All patients	Endpoint (*n* = 13)	No endpoint (*n* = 87)	*P*
Applications on PW	30 [24–36]	32 [26.1–37.9]	29 [24–36]	0.31
Mean ILD on PW, mm	4.09 [3.71–5.35]	3.76 [3.54–4.23]	4.16 [3.74–5.51]	**0**.**04**
Minimum ILD on PW, mm	1.4 [0.9–2]	1.42 [0.77–2.07]	1.4 [0.9–2.05]	0.58
Longest application on PW, s	11.2 [4.05–18.5]	12.0 [4.06–16.2]	10.5 [4.05–18.6]	0.89
Mean application time on PW, s	4.18 [3.97–10.7]	4.22 [3.99–10.5]	4.14 [3.97–10.7]	0.65
Maximal catheter tip temperature on PW, °C	52 [49–57]	52.4 [47.5–57.2]	52 [49–57]	0.68
Mean catheter tip temperature on PW, °C	42.3 [40.2–45.5]	43.0 [39.1–47.0]	42.2 [40.3–45.5]	0.97
Mean CF during energy delivery on PW, g	15.6 [12.2–20.2]	14.3 [10.2–18.5]	15.9 [12.4–20.4]	0.13
Maximal CF during energy delivery on PW, g	83.6 [57.0–110]	72.5 [48.1–97.0]	85.4 [58.7–112]	0.09
Mean power, W	59.4 [46.2–82.1]	77.6 [46.1–82.8]	48.8 [46.3–82.0]	0.80
Total energy delivered on PW (J)	9786 [7492–12 407]	10 667 [6403–14 930]	9794 [7492–12 120]	0.64
Oesophagus-LA contact length, mm	40.0 [31.0–48.9]	36.6 [26.4–46.9]	40.5 [31.7–49.2]	0.24
PW min. thickness, mm	0.66 [0.57–0.73]	0.66 [0.57–0.75]	0.66 [0.57–0.73]	0.72
Oesophagus wall min. thickness, mm	3.29 [2.44–4.14]	3.06 [2.21–3.91]	3.32 [2.47–4.17]	0.36
Fat pad thickness, mm	0.57 [0–0.83]	0.47 [0.31–0.78]	0.63 [0–0.86]	0.83
Endocardium-oesophageal min. distance, mm	0.9 [0.6–1.2]	1.05 [0.51–1.59]	0.9 [0.6–1.2]	0.68
Endocardium-oesophageal mucosa min. distance, mm	4.41 [3.36–5.46]	4.34 [3.2–5.48]	4.42 [3.38–5.46]	0.90

Bold *P* values indicate statistically significant difference.PW, posterior wall; CF, contact force; LA, left atrium.

## Discussion

The main finding of this study is that PVI using the vHPSD power setting is non-inferior to the HPSD setting regarding the incidence of oesophageal erosions and gastric hypomotility. Furthermore, a smaller mean ILD on the posterior wall was associated with a higher incidence of thermal injury. Although some prior studies have examined this issue, they either concentrated solely on HPSD ablation^14,16,22,26–28^ or vHPSD ablation,^26^ were retrospective in nature,^29^ or did not require mandated post-procedural endoscopy.^27^ In contrast, the present study offers a direct comparison of the two ablation modalities, with a focus on assessing thermal safety.

### Very high power short duration ablation method

Very high power short duration technology offers similar efficacy to lower energy modalities while enabling a faster workflow. This advantage in time efficiency is increasingly important in the era of pulsed-field ablation (PFA), a new technology offering unprecedented reduction in procedure times, with comparable success rates. It is important to note that vHPSD per definition only refers to the ablation at 90 W for 4 s. The procedure requires a dedicated catheter (e.g. QDot Micro), as delivering such a high power requires a rapid and precise temperature control algorithm (QMODE+).^30^ It has recently been the focus of extensive research on how increased RF power affects lesion properties. Initial studies proposed that vHPSD energy may produce shallower but larger lesions.^31^ Later, *ex vivo* experiments confirmed that lesions are indeed shallower but also smaller in area and total volume.^10^ Interestingly, while surface temperatures were higher, temperature rise in deeper tissue layers of the tissue was the lowest in the case of 90 W/4 s applications, indicating a reduced tissue penetration. Favourable *in vivo* lesion metrics, shorter procedure times, and a similar efficacy lead to the increased use of the 90 W power setting. A recent registry study compared the use of vHPSD ablation on the posterior wall to purely AI-guided, non-vHPSD ablation. Both groups had continuous oesophageal temperature monitoring. The authors found that the vHPSD group had a lower incidence of a temperature rise above 40 degrees in the oesophagus, suggesting that this power setting offers better thermal safety.^32^ Nevertheless, there was no previous randomized study comparing its oesophageal safety to lower power modalities.

### Endoscopy findings

Previous data suggests that vHPSD may have a reduced impact on surrounding tissues, including the oesophagus and the adjacent vagal fibres.^10^ Our results also support this concept by showing that both the HPSD and the vHPSD setting result in a low incidence of thermal injury; however, it also remains clear that these complications may still occur despite precautions when using a thermal energy source. Compared to previous data, we found similar rates of oesophageal erosions with HPSD, while the two erosions per 50 patients in the vHPSD arm slightly exceeded the incidence that was reported in previous studies.^14^ The QDot FAST trial reported one oesophageal ulcer among 52 patients, though this study did not include mandated post-procedural endoscopy.^7^ Later, Halbfass *et al*. reported one erosion in 90 patients who underwent endoscopy following vHPSD PVI.^29^ More recently, in a randomized study comparing 30/50 W and 90 W ablation, a subgroup of patients underwent post-operative endoscopy. In the total of 55 (30/50 W) and 57 (90 W) patients, one oesophageal ulcer was noted in the 50 W group, while one erosion was found in the 90 W group.^32^ Gastric hypomotility was used as part of the primary endpoint due to its association with vagal injury, which may result from the ablation of the oesophageal adventitial layer. Importantly, there is ample literature on vagal injury and consequent post-operative gastric hypomotility in the case of HPSD PVI,^33^ often considered as a surrogate marker to thermal injury to the vagus nerve.^33^ Nevertheless, to date, there is no detailed report on this aspect regarding vHPSD energy. Here, we demonstrate a comparable, non-negligible rate of gastroparesis in the vHPSD group, emphasizing the importance of awareness of vagal nerve complications with this power setting.

In this context, the multicentre Spanish POWER-FAST III randomized trial compared an HPSD strategy using 70 W for 9–10 s with conventional power delivery (25–40 W) and mandated post-procedural endoscopy.^34^ Endoscopically detected oesophageal lesion occurred in 3.6% with 70 W and 2.7% with 25–40 W (*P* = 0.94). The low incidence of EDEL, comparable between arms, mirrored our observations (4% with 90 W vs. 2% with 50 W), albeit our primary endpoint additionally included gastroparesis and thus yielded higher composite rates (14% vs. 12%). Notably, POWER-FAST III delivered 70 W using conventional contact-force–sensing open-irrigated catheters rather than a dedicated temperature-controlled vHPSD catheter, and therefore, its findings are not directly generalizable to contemporary 90 W/4 s vHPSD workflows. Moreover, current consensus documents typically frame HPSD around 45–50 W and vHPSD around 90 W/4 s, whereas 70 W/9–10 s remains less widely adopted in routine practice, which downplays the applicability of POWER-FAST III to our study design and technology.^3^

### Ablation parameters

The quality of point-by-point PVI is known to depend on lesion quality and density. Lesion quality depends on several factors, including CF, time, and power; HPSD ablation systems often combine these parameters to calculate lesion indices, such as the AI.^35^ Another important factor, lesion density, is often expressed as the average ILD. The widely accepted and validated CLOSE protocol defines a maximal ILD of 6 mm and an AI of 400 at the posterior wall and 550 at the anterior wall.^36^ Since the 90 W vHPSD system utilizes standard 4 s applications, a modified version of the CLOSE protocol was developed involving 3–4 mm ILD at the anterior aspect and 5–6 mm at the posterior aspect of the left atrium, yielding excellent results.^27^ Furthermore, our group has recently confirmed that a larger ILD predicts local reconnections 3 months after the procedure.^36^

Analysing the correlation between ablation parameters and the primary outcome, we found that a mean ILD of 3.76 mm is associated with a higher incidence of thermal injury. We believe that ILD is of special importance with the vHPSD technology, which uses uniform 4 s applications, enabling less control over lesion depth. Importantly, while vHPSD employs real-time energy titration based on tissue temperature,^22,36^ ‘thermal latency’ still remains an issue, implying that tissue temperatures continue to rise after RF termination.^10^ This effect may result in an additive effect of consecutive applications, which may be further aggravated by a smaller ILD. Our observations suggest that additionally to keeping a brief pause between applications in the vicinity of the oesophagus, avoiding unnecessary overlap between applications (e.g. avoiding too short ILD values) might mitigate thermal injury. Importantly, while 3.76 mm mean ILD on the posterior wall may seem small, one must consider that in the case of a target ILD of 5 mm, the actual ILD often approximates to 3.5–4 mm.^22,37^

Our findings regarding ILD align with a recent study, which found that a smaller mean distance between the left and right PVI circles predicted thermal injury.^11,14,15,38^ This study also showed that in patients with lesions, the mean CF on the posterior wall was higher, which associates with higher maximal ablation temperatures.^30^ Interestingly, we did not observe a correlation between the endpoint and CF or catheter tip temperatures. This may be attributed to the facts that the mean CF was well within the safety margins (17.7 g with vHPSD and 14.2 g) with HPSD and that catheter tip temperature was accurately measured by the QDot Micro catheter and closely controlled in real time by the ablation system.

### Anatomical factors

We analysed pre-operative CT angiography images for anatomical factors predisposing to thermal injury. Most previous studies assessed the minimal distance between the oesophageal mucosa and the LA endocardium, and this parameter was often identified as a predictor of thermal injury.^22,37^ While our measurements were consistent with previously reported data, we found no correlation between this parameter and the occurrence of our endpoint. Furthermore, we performed a series of additional measurements, including the thickness of the oesophagus, the atrial posterior wall, and the fat pad between the two structures; however, none of these were predictive of thermal injury. We propose two possible explanations to this discrepancy. First, our composite endpoint also included gastroparesis, which may depend less on atrial-oesophageal distance. Second, we observed a significantly lower incidence of thermal mucosal injury, leading to less statistical power.

### Preventive measures

Albeit uncommon, oesophageal perforation remains a critical concern in modern thermal ablation. Numerous approaches to oesophageal protection have been evaluated in the literature. The use of an oesophageal temperature probe was evaluated in multiple randomized studies, including one with an HPSD power setting, yet no clear benefit was found.^15,38,39^ Similarly, proactive oesophageal cooling has shown only marginal benefits in reducing the severity of thermal injury.^40^ Given the uncertainty regarding these protective strategies, our study protocol did not mandate oesophageal temperature monitoring or cooling. The low frequency and self-limiting nature of thermal injuries observed in our study support the view that both energy settings present only a minimal risk.

## Conclusion

Pulmonary vein isolation using vHPSD (90 W, 4 s) applications was non-inferior to the 50 W power setting in the context of thermal injury, defined as the composite of post-ablation oesophageal lesions and gastric hypomotility. A smaller ILD on the posterior wall may negatively impact oesophageal safety.

## Limitations

This trial has some limitations to be acknowledged. First, while EDEL is an established surrogate for AEF, most EDELs are mild and self-limiting, and the predictors of progression to clinically meaningful injury or AEF remain uncertain. In addition, detection and grading are subject to inter-observer variability and methodological differences. Furthermore, while gastric hypomotility is an established marker of vagal thermal injury, its clear association with AEF formation remains to be shown. Certainly, a substantially larger sample size would allow the comparison of the two modalities with regard to mucosal injury alone. Second, the post-ablation endoscopy was performed within 15 days. This is a longer time window compared to many previous studies,^11,14,15,41^ although some studies used similar timing.^8^ Consequently, the incidence of mild oesophageal lesions may be underestimated. However, we believe that lesions with a tendency to heal within days have little relevance regarding the risk of AEF formation. Third, we did not monitor the position of the oesophagus during the procedure. There is anatomical variation in the position of the oesophagus relative to the LA PW which can affect its proximity to the actual ablation lines. In this study, we assessed the properties of all RF applications on the PW, regardless of their position relative to the oesophagus. Moreover, we measured the distance between the oesophagus and their closest proximity, regardless of the relative position of the applications. These factors may have limited our assessment of procedural and anatomical predictors of thermal injury. Finally, although all patients underwent pre-procedural CT and ablation data were available in every case, the modest endpoint incidence limited statistical power and precluded reliable multiple regression. Therefore, the predictors of EDEL were only assessed using univariate analysis. Accordingly, any associations found should be interpreted with caution as hypothesis-generating.

## Supplementary Material

oeag041_Supplementary_Data

## Data Availability

The data underlying this article cannot be shared publicly due to privacy/ethical reasons (General Data Protection Regulation). The data will be shared on reasonable request to the corresponding author.
