# Supplementary material for: Oesophageal safety of high and very high power short duration pulmonary vein isolation: a randomized comparison of the 50 W and 90 W power settings—the HPSD oesophagus study
Source: Eur Heart J Open. 2026 Feb 25;6(2):oeag041. doi: 10.1093/ehjopen/oeag041 (PMC13033446; doi:10.1093/ehjopen/oeag041)
Supplement: oeag041_Supplementary_Data [file oeag041_supplementary_data.docx]

**Esophageal Safety of High and Very High Power Short Duration Pulmonary Vein Isolation: a Randomized Comparison of the 50W and 90W power settings**

**Supplemental methods**

**Inclusion criteria**

- - Symptomatic paroxysmal/persistent AF
  - Age >18 years
  - Willingness to sign informed consent form

**Exclusion criteria**

- - Contraindication to ablation
  - Contraindication of long-term anticoagulation
  - Long-standing persistent AF
  - History of PVI
  - History of cardiac surgery
  - Pregnancy
  - Active malignancy
  - Life expectancy <1 year
  - Valvular AF
  - Reversible cause of AF (e.g. hyperthyroidism)
